# High prevalence but lack of awareness of hypertension in South Africa, particularly among men and young adults

**DOI:** 10.1038/s41371-023-00873-3

**Published:** 2023-10-25

**Authors:** Angela J. Woodiwiss, Ane Orchard, Catharina M. C. Mels, Aletta S. Uys, Benedicta N. Nkeh-Chungag, Andrea Kolkenbeck-Ruh, Lisa J. Ware, Samantha Yates, Erika S. W. Jones, Vernice R. Peterson, Neil R. Poulter

**Affiliations:** 1 Cardiovascular Pathophysiology and Genomics Research Unit, School of Physiology, Faculty of Health Sciences, University of the Witwatersrand, Johannesburg, South Africa; 2 Clinical Pharmacy Division, Department of Pharmacy and Pharmacology, Faculty of Health Sciences, University of the Witwatersrand, Johannesburg, South Africa; 3 Hypertension in Africa Research Team (HART), Faculty of Health Sciences, North-West University, Potchefstroom, South Africa; 4 SAMRC Research Unit for Hypertension and Cardiovascular Disease, Faculty of Health Sciences, North-West University, Potchefstroom, South Africa; 5 Department of Biological and Environmental Sciences, Faculty of Natural Science, Walter Sisulu University, Mthatha, South Africa; 6 SAMRC-Wits Developmental Pathways to Health Research Unit, Faculty of Health Sciences, University of the Witwatersrand, Johannesburg, South Africa; 7 Department of Life and Consumer Sciences, University of South Africa (UNISA), Science Campus, Roodepoort, South Africa; 8 Division of Nephrology and Hypertension, University of Cape Town, Cape Town, South Africa; 9 Imperial Clinical Trials Unit, Imperial College London, Stadium House, 68 Wood Lane, London, W12 7RH UK

**Keywords:** Diseases, Health care

## Abstract

Cardiovascular disease (CVD) is a leading cause of death in South Africa (SA) and high blood pressure (BP) is the primary risk factor. However, hypertension prevalence is high, BP control is poor and CV events occur at a younger age than in Europe or America. Increasing screening, raising awareness and improving management of hypertension are critical to prevent CVD in SA. May Measurement Month (MMM) is a global initiative of the International Society of Hypertension aimed at raising awareness of high BP. As part of the MMM campaign, in SA (2017, 2018, 2019 and 2021), BP measurements and a cross-sectional survey of volunteers aged ≥ 18years were performed. Of 11,320 individuals (age 36.6 ± 16.8years) screened, 29.7% had hypertension (systolic BP/diastolic BP ≥ 140/90 mmHg or antihypertensive medication use) and the prevalence was higher (*p* < 0.0001) in men (35.6%) than in women (26.3%). Of those with hypertension, only 54.3% were aware and 46.8% were receiving antihypertensive medication, and 53.7% of these had controlled BP. In men with hypertension, awareness (45.2%, treatment (38.2%) and controlled BP on antihypertensive medication (45.2%) were lower (*p* < 0.0001) than in women (awareness: 60.8%; treatment: 53.5%; controlled BP: 58.3%). In young participants (age < 40years), 15.6% had hypertension, 18.6% of these were on treatment but 76.0% were not aware, and only 57.7% had controlled BP. The high prevalence of hypertension, but low levels of awareness, treatment, and BP control in SA, especially in young adults and men, highlight the need for systematic BP screening programmes and improvements in education and management of hypertension.

## Introduction

Cardiovascular disease (CVD) continues to be a leading cause of death in South Africa, with one in every six deaths being attributed to CVD [1]. High blood pressure (BP) is the single risk factor that explains most population-attributable risk for CVD [2]. In sub-Saharan Africa the prevalence of hypertension amongst adults is higher and the proportion of individuals achieving BP control is lower than in other regions of the world [3, 4]. Furthermore, in sub-Saharan Africa cardiovascuar events occur in individuals ~15 years younger than in other populations globally [3, 5]. National surveys in South Africa have indicated a hypertension prevalence ranging from 38.4% in 2012 (the South African National Health and Nutrition Examination Survey [SANHANES]) [6] to 48.2% in 2016 (the Demographic and Health Survey [DHS]) [7]. Moreover, at least one-third of hypertensive individuals were unaware of their hypertension status and only a quarter achieved BP control on anti-hypertensive medication [8]. Consequently, raising awareness of hypertension, increasing screening for hypertension and improving the management of hypertension are critical to prevent CVD in South Africa. Hence, in 2017, when the BP screening campaign (May Measurement Month, MMM) was instituted globally, South Africa became involved [9]. Free opportunistic BP screening has continued in South Africa in each subsequent year, except for in 2020 when as with other countries globally, MMM activities were halted due to the global COVID-19 pandemic. The results of MMM data collected in South Africa from 2017 to 2021 are presented in this paper.

## Methods

### Study group

The present study was approved by the Committee for Research on Human Subjects of the University of the Witwatersrand (M170334); the Health Research Ethics Committee of the North-West University (NWU-00026-17-A5); the College of Agriculture and Environmental Sciences Research Ethics Review Committee of the University of South Africa (UNISA) (2018/CAES/062); the Human Research Ethics Committee of Walter-Sisulu University (030/2018); the Human Research Ethics Committee, Faculty of Health Sciences, University of Cape Town (FWA00001637, IRB00001938); and the Human Sciences Research Council (10/22/03/17). Participants gave written informed consent.

In total, 13,236 participants aged 18 years and older were screened at the University of the Witwatersrand (Parktown, East and West campuses), in Johannesburg, Gauteng; the North-West University (Potchefstroom campus); Ikageng Gate Shopping Centre in Potchefstroom, North West Province; the Moletsane Sports Complex and surrounding households in SOWETO, Gauteng; the Walter Sisulu Botanical Gardens in Roodepoort, Gauteng; the Bryanston Organic Market in Johannesburg, Gauteng; the Groote Schuur Hospital in Cape Town, Western Cape; the Batlhabine Traditional Area, Tzaneen, Mpumalanga; the BT Ngebs Shopping Centre in Mthatha, Eastern Cape; King Edward VIII Hospital, Durban, KwaZulu Natal; various community centres in Potchefstroom, North West Province; a community clinic in Vosloorus, Gauteng; various physiotherapy practices, health care centres and schools in Johannesburg, Gauteng; the Ngwathe Local Municipality Area, Vredefort, Free State; Dischem retail pharmacies and various other retail and community pharmacies throughout the country. Screening was primarily on weekdays during the months of May to November. Participants were screened at variety of locations including university campuses, shopping malls, community centres, community recreational areas, pharmacies, biokinetics and physiotherapy practices, community clinics and hospital clinics. Of the 13 236, 3 250 (24.6%) were screened in 2017, 2965 (22.4%) in 2018, 4727 (35.7%) in 2019 and 2294 (17.3%) in 2021. Due to the COVID-19 global pandemic, screening was halted in 2020.

### Blood pressure, demographic and anthropometric measurements

Before the commencement of data collection, volunteers were trained in accurate blood pressure (BP) measurement techniques using validated automated devices (Omron MIT5 Connect and Omron M6 Comfort devices, Omron Healthcare). At least three seated BP and heart rate recordings were taken on the left arm (preferably) after at least 5 min rest with 1-min intervals. A questionnaire was used to collect limited social, clinical, lifestyle and demographic data as previously described [10]. Those participants whose BP was potentially in the hypertensive range (systolic BP [SBP] ≥140 mmHg and/or diastolic BP [DBP] ≥90 mmHg) were given non-pharmacological and lifestyle advice and were also advised to have their BP rechecked with a time scale depending on the degree of BP elevation. The data were entered either directly onto a bespoke mobile application or into a study-specific worksheet (Excel). The data were cleaned by each of the site principal investigators before submitting them to MMM central. 11,320 participants had all 3 blood pressure readings available for analyses. Data on awareness of hypertension was not collected in 2017.

### Data analysis

Analyses were done using the mean of the second and third BP readings. The diagnosis of hypertension was based upon a mean SBP ≥ 140 mmHg and/or DBP ≥ 90 mmHg or receiving treatment for hypertension. Proportions of all participants with hypertension, aware of their hypertension and receiving treatment for their hypertension were calculated. Proportions were also calculated in various sub-groups: hypertensives; women; men; decades of age; various ethnicities (self-identification) and different provinces in South Africa. The effects of the sub-groups on the various proportions were assessed using Chi-square analysis. Controlled BP was defined as SBP/DBP < 140/90 mmHg.

## Results

### General characteristics

The general characteristics of the 11 320 participants screened are shown in Table 1. The majority of the participants were young, with 46.0% being <30 years of age and only 12.9% being ≥60 years of age. Approximately two-thirds of the participants were female and less than a quarter currently smoked or reported frequent alcohol intake. The proportions with diabetes, previous myocardial infarction or stroke were low, as were the percentages using aspirin or statins. The participants were predominantly of black or white ethnicity, and the majority were screened in either the North West Province or Gauteng. Although 47.6% of participants weighed more than 70 kg, 59.2% met the WHO physical activity guidelines. Only 7.0% had previously participated in an MMM campaign.

**Table 1:** Characteristics of the participants screened.

Sample number (% female)	11,320 (63.7)
Age (years)	36.6 ± 16.8
% Age < 30years (*n*)	46.0 (5207)
% Age ≥ 60years (*n*)	12.9 (1460)
Body weight (kg)	73.8 ± 17.4
Body height (kg)	166.2 ± 9.9
Body mass index (kg/m^2^)	26.7 ± 6.1
% Current smoker	14.6
% Regular alcohol intake^a^	22.6
% Diabetes mellitus	3.9
% Pregnant women	1.8
% Previous myocardial infarction	1.4
% Previous stroke	1.2
% Receiving aspirin	11.9
% Receiving statin	9.0
% Meeting WHO physical activity guidelines	59.2
Ethnicity	
% Black (*n*)	60.2 (6 814)
% White (*n*)	30.3 (3 430)
% Mixed (*n*)	3.6 (408)
% South Asian (*n*)	2.3 (261)
% East Asian (*n*)	0.3 (34)
% Arabic (*n*)	0.3 (34)
% South-east Asian (*n*)	0.3 (34)
% Other (*n*)	2.3 (260)
% Unknown^b^ (*n*)	0.4 (45)
Province	
% North West Province (*n*)	41.21 (4665)
% Gauteng (*n*)	38.81 (4393)
% Eastern Cape (*n*)	13.44 (1521)
% Limpopo (*n*)	2.92 (331)
% Western Cape (*n*)	1.99 (225)
% Free State (*n*)	1.48 (168)
% KwaZulu Natal (*n*)	0.07 (8)
% Mpumalanga (*n*)	0.07 (8)
% Northern Cape (*n*)	0.01 (1)

^a^Regular alcohol intake defined as once or more per week.

^b^Not recorded.

### Blood pressure and heart rate values

Table 2 shows the BP and heart rate readings in all participants, and in men and women separately. The first BP (SBP/DBP) readings were consistently higher than the second or third readings. In all participants, the first SBP reading was 2.8 ± 8.7 mmHg higher (*p* < 0.0001) than the second SBP reading, and 4.2 ± 9.0 mmHg higher (*p* < 0.0001) than the third SBP reading. In addition, in all participants, the first DBP reading was 2.0 ± 7.4 mmHg higher (*p* < 0.0001) than the second DBP reading, and 2.9 ± 7.4 mmHg higher (*p* < 0.0001) than the third DBP reading. Treated participants had higher SBP and DBP than untreated participants (Table 2), consistent with only a half of treated hypertensives having controlled blood pressure (Table 3). Men had higher SBP and DBP than women (Table 2).

**Table 2:** Blood pressure and heart rate values of the participants screened.

	All Participants	Treated Participants	Excluding Treated	Women	Men
(*n*)	(11,320)	(1573)	(9747)	(7211)	(4109)
SBP reading 1 (mmHg)	125 ± 19	139 ± 21	123 ± 17	121 ± 18	132 ± 17
SBP reading 2 (mmHg)	122 ± 18	135 ± 20	120 ± 16	119 ± 17	129 ± 17
SBP reading 3 (mmHg)	121 ± 17	134 ± 20	119 ± 16	117 ± 17	128 ± 16
SBP readings 1, 2 and 3 (mmHg)	123 ± 17	136 ± 20	121 ± 16	119 ± 17	130 ± 16
SBP readings 2 and 3 (mmHg)	122 ± 17	135 ± 20	120 ± 16	118 ± 17	128 ± 16
DBP reading 1 (mmHg)	82 ± 12	88 ± 13	81 ± 11	82 ± 12	84 ± 12
DBP reading 2 (mmHg)	80 ± 12	86 ± 13	79 ± 11	80 ± 11	82 ± 12
DBP reading 3 (mmHg)	79 ± 11	85 ± 12	79 ± 10	79 ± 11	81 ± 12
DBP readings 1, 2 and 3 (mmHg)	81 ± 11	86 ± 12	80 ± 10	80 ± 11	82 ± 11
DBP readings 2 and 3 (mmHg)	80 ± 11	86 ± 12	79 ± 11	79 ± 11	81 ± 11
HR reading 1 (bts/min)	77.2 ± 13.2	77.7 ± 13.2	77.2 ± 13.3	79.3 ± 12.7	73.6 ± 13.5
HR reading 2 (bts/min)	77.0 ± 13.0	77.2 ± 13.0	77.0 ± 13.0	78.9 ± 12.5	73.5 ± 13.1
HR reading 3 (bts/min)	77.2 ± 12.9	77.1 ± 13.0	77.2 ± 12.9	79.1 ± 12.5	73.9 ± 13.1
HR readings 1, 2 and 3 (bts/min)	77.1 ± 12.5	77.3 ± 12.7	77.0 ± 12.4	79.0 ± 11.9	73.6 ± 12.7

*SBP* systolic blood pressure, *DBP* diastolic blood pressure, *HR* heart rate.

**Table 3:** Proportions of participants with hypertension, treated for hypertension, aware of hypertension and having controlled blood pressure.

	All participants	Women	Men
*n*	11,320	7211	4109
% Hypertension	29.7	26.3	35.6*
% Treated	13.9	14.0	13.6
% of HT treated	46.8	53.5	38.2*
% of HT controlled	25.2	31.2	17.3*
% of HT treated & controlled	53.7	58.3	45.2*
*n*^a^	8 144	5 163	2 981
% Hypertension	32.3	29.7	36.8*
% Treated	15.5	16.2	14.3
% of HT treated	47.9	54.5	38.8*
% Aware	20.4	21.3	18.9
% of HT aware	54.3	60.8	45.2*
% of HT controlled	25.6	31.0	18.0*
% of HT treated & controlled	53.3	56.9	46.2*

Controlled, SBP/DBP < 140/90 mmHg.

*HT* hypertension.

*p < 0.0001 versus women.

^a^Excluding participants screened in 2017 as awareness of hypertension was not assessed.

### Proportions with hypertension, treatment, awareness of hypertension and BP control

The data in Table 3 are shown for whole cohort (*n* = 11,320) as well as the cohort excluding participants screened in 2017 (*n* = 8144) as awareness of hypertension was not assessed in the first year of the MMM campaign (2017). Importantly, the proportions with hypertension, treatment and BP control did not differ between the two cohorts (Table 3). Almost a third of the participants were hypertensive (Table 3), with more men than women being hypertensive (Table 3), despite being of the same mean age (men: 36.96 ± 16.79 years; women: 36.44 ± 16.85 years; *p* = 0.12). Only 7.5% of women reported having hypertension during pregnancy. Less than half of hypertensives were receiving treatment for hypertension (Table 3), with the proportion being lower in men compared to women. Approximately, only a half of hypertensives were aware of their condition and again this proportion was lower in men compared to women (Table 3). Only half of the treated hypertensives had controlled BP (<140/90 mmHg), with the proportion being lower in men compared to women (Table 3).

### Impact of age on proportions with hypertension, treatment, aware of hypertension and BP control

**Fig. 1:**
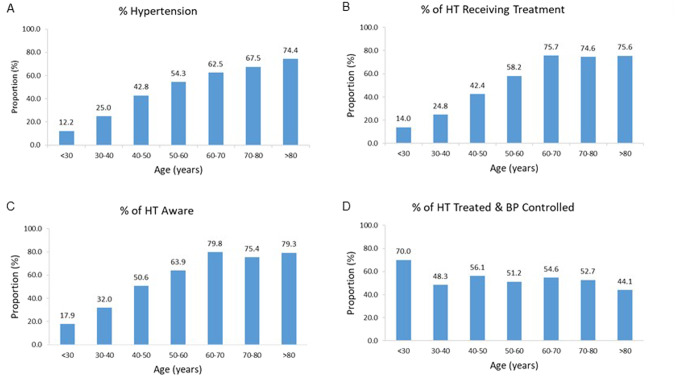
Impact of age on proportions of all participants with hypertension, and on proportions of hypertensives receiving treatment for hypertension, aware of their hypertension and with controlled blood pressure. Proportions of all participants with hypertension (**A**), and proportions of hypertensive participants (HT) who were receiving treatment for hypertension (**B**), or were aware of their hypertension (**C**), or had controlled blood pressure (BP) per decade of age. *P* < 0.0001 for effect of decade of age. *n* for each decade of age = 5269, 1901, 1493, 1198, 889, 449, 121, respectively for (**A**); 645, 475, 639, 651, 556, 303, 90 for (**B**); 441, 337, 470, 543, 471, 280, 87 for (**C**); and 90, 118, 271, 379, 421, 226, 68 for (**D**).

The proportions of participants with hypertension increased with each increment in decade of age, with the most marked increases noted up to the age of 60 years (Fig. 1A). Below 60 years of age the proportions of hypertensive individuals who were receiving treatment for their hypertension declined with each decrease in decade of age (Fig. 1B). In addition, the proportions of hypertensive individuals who were aware of their condition declined with each decrease in decade of age below 60 years (Fig. 1C). However, over the age of 60 years more than three-quarters of the participants with hypertension were receiving treatment for their hypertension (Fig. 1B) and approximately 80% were aware of their condition (Fig. 1C). The proportions of individuals with hypertension receiving treatment and achieving controlled BP was similar across the decades of age, except for in those who were <30 years of age where the proportion was higher (Fig. 1D). In young participants (age < 40 years), 15.6% had hypertension and 18.6% of these were receiving antihypertensive medication. Only 24.0% of young participants with hypertension were aware of their hypertensive status and only 57.7% of young participants who were receiving antihypertensive medication had controlled BP.

### Impact of gender and age on proportions with hypertension, treatment, aware of hypertension and BP control

**Fig. 2:**
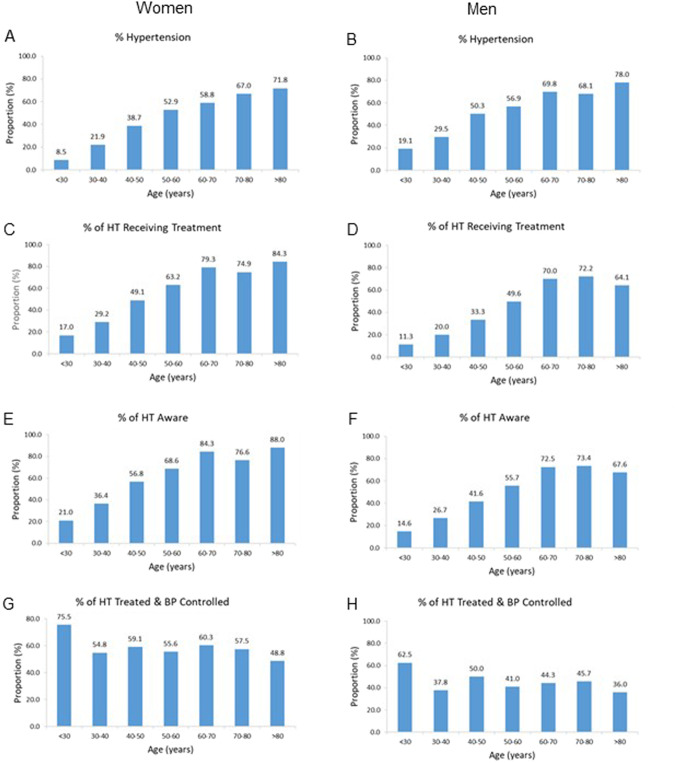
Impact of gender on proportions of all participants with hypertension, and on proportions of hypertensives receiving treatment for hypertension, aware of their hypertension and with controlled blood pressure. Proportions of all women and all men with hypertension (**A**, **B**), and proportions of hypertensive participants (HT) who were receiving treatment for hypertension (**C**, **D**), or were aware of their hypertension (**E**, **F**), or had controlled blood pressure (BP) (**G**, **H**) per decade of age. In women n for each decade of age = 3409, 1141, 955, 782, 584, 268, 72, respectively for (**A**); 289, 250, 369, 413, 343, 179, 51 for (**C**); 200, 187, 280, 347, 293, 171, 50 for (**E**); and 49, 73, 181, 261, 272, 134, 43 for (**G**). In men *n* for each decade of age = 1852, 763, 538, 416, 306, 183, 51, respectively for (**B**); 354, 225, 270, 236, 213, 124, 39 for (**D**); 239, 150, 190, 194, 178, 109, 37 for (**F**); and 40, 45, 90, 117, 149, 92, 25 for (**H**).

As with all individuals, the proportions of all women and all men with hypertension increased with each increment in decade of age, with the most marked increases noted up to the age of 60 years (Fig. 2A, B). The proportions of men who had hypertension were consistently greater than that of women (Fig. 2A, B). Below 60 years of age the proportions of hypertensive individuals who were receiving treatment for their hypertension declined with each decrease in decade of age (Fig. 2C, D). At each decade of age, the proportion of men who were receiving treatment for their hypertension was consistently lower than that of women. In addition, the proportions of hypertensive individuals who were aware of their condition declined with each decade of age below 60 years (Fig. 2E, F), with consistently lower proportions in the men than in the women (Fig. 2E, F). The proportions of hypertensive women receiving treatment and achieving controlled BP was similar across the decades of age, except for those who were <30 years of age where the proportion was higher (Fig. 2G). The proportions of hypertensive men receiving treatment and achieving controlled BP were consistently lower than in women across the decades of age (Fig. 2G, H), but were fairly consistent across the decades of age, except for in those who were <30 years of age where the proportion was higher (Fig. 2H).

### Impact of ethnicity on proportions with hypertension, treatment, aware of hypertension and BP control

**Fig. 3:**
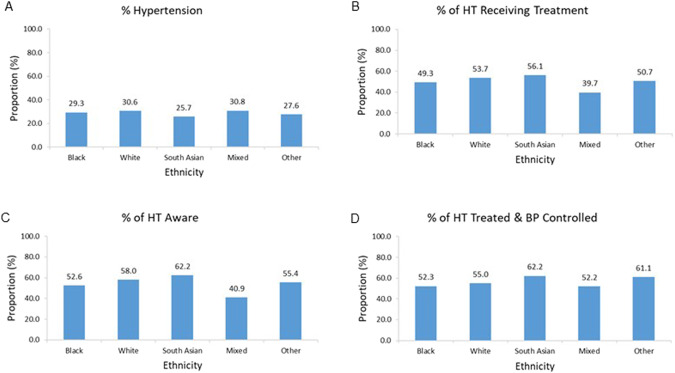
Impact of ethnicity on proportions of all participants with hypertension, and on proportions of hypertensives receiving treatment for hypertension, aware of their hypertension and with controlled blood pressure. Proportions of all participants with hypertension (**A**), and proportions of hypertensive participants (HT) who were receiving treatment for hypertension (**B**), or were aware of their hypertension (**C**), or had controlled blood pressure (BP) (**D**) per ethnic group. *P* = 0.046 for effect of ethnic group on treatment; *P* = 0.005 for effect of ethnic group on awareness. *n* for each ethnicity = 6764, 3408, 261, 403, 257, respectively for (**A**); 1982, 1041, 67, 124, 71 for (**B**); 1588, 793, 45, 93, 56 for (**C**); and 886, 524, 37, 46, 36 for (**D**).

The data shown are only for those ethnic groups with over 200 participants. The proportions with hypertension were fairly consistent (*p* = 0.43 for the effect of ethnicity) across the various self-identified ethnic groups (Fig. 3A), with South Asian’s having the lowest proportion and those of mixed ancestry the highest (Fig. 3A). There was a marginal effect of ethnicity (*p* = 0.046) on the proportions of participants with hypertension receiving treatment for their hypertension, with those of mixed ancestry having a lower proportion than in the other groups (Fig. 3B). The proportion of participants with hypertension aware of their condition was similar amongst blacks and whites, with South Asian’s having the highest proportion and those of mixed ancestry the lowest (Fig. 3C). The proportions of participants with hypertension receiving treatment and achieving controlled BP was similar (*p* = 0.56) across ethnic groups, with South Asian’s having the highest proportion and those of mixed ancestry the lowest (Fig. 3D).

### Impact of province on proportions with hypertension, treatment, aware of hypertension and BP control

**Fig. 4:**
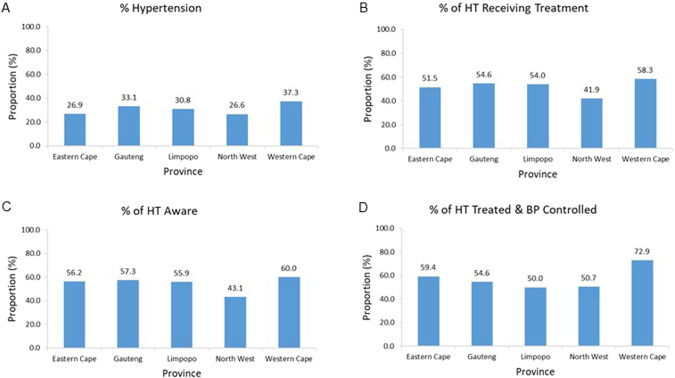
Impact of province on proportions of all participants with hypertension, and on proportions of hypertensives receiving treatment for hypertension, aware of their hypertension and with controlled blood pressure. Proportions of all participants with hypertension (**A**), and proportions of hypertensive participants (HT) who were receiving treatment for hypertension (**B**) or were aware of their hypertension (**C**), or had controlled blood pressure (BP) (**D**) per province. *P* < 0.0001 for effect of province. *n* for each province = 1521, 4393, 331, 4665, 225 respectively for (**A**); 409, 1453, 102, 1194, 84 for (**B**); 409, 1303, 102, 626, 80 for (**C**); and 192,720, 54, 487, 48 for (**D**).

The data shown are only for those provinces with over 200 participants. The proportions with hypertension varied (*p* < 0.0001) across the provinces (Fig. 4A), being the lowest in the North West and the highest in the Western Cape (Fig. 4A). Similarly, the proportions of individuals with hypertension receiving treatment for their hypertension varied (p < 0.0001) across the provinces, with the proportion in the North West province being lower than in the other provinces (Fig. 4B). The proportion of individuals with hypertension aware of their condition also varied (*p* < 0.0001) across the provinces, with again the proportion being the lowest in the North West province (Fig. 4C). The proportions of individuals with hypertension receiving treatment and achieving controlled BP varied (*p* = 0.02) across the provinces, with the Western Cape having the highest proportion (Fig. 4D).

## Discussion

In summary, the data show that amongst an opportunistic sample of adults in South Africa, the prevalence of hypertension is high (~30%) despite the low mean age (<40 years). However, the proportions of individuals with hypertension receiving treatment for hypertension and aware of their hypertension were low (<50%). Consequently, the proportion of participants with hypertension who were treated and who had controlled BP was also low (~50%). More men than women were hypertensive, and the proportions of hypertensives receiving treatment for hypertension, aware of their hypertension and with controlled BP were lower in men than in women. Ethnicity had little impact on the proportion with hypertension, but treatment, awareness and the proportion treated with controlled BP were the lowest amongst those of mixed ancestry. Hypertension prevalence was the highest in the Western Cape, but the proportions of individuals with hypertension receiving treatment for hypertension, aware of their hypertension and the proportion treated with controlled BP were also the highest in this province. The proportions of individuals with hypertension receiving treatment for hypertension and aware of their hypertension were the lowest in the North West Province.

The prevalence of hypertension in an opportunistic sample of adults in South Africa is similar to that reported globally (34%); but the proportions of hypertensives receiving antihypertensive medication or who were aware of their diagnosis of hypertension were lower than those reported globally (medication: 54.7%; aware: 58.7%) [11]. Nevertheless, the proportion of South African participants with hypertension who were receiving antihypertensive treatment and had controlled BP was similar to that reported globally (57.8%) [11]. The proportions of South Africans with hypertension, aware of their hypertension and having controlled BP on antihypertensive medication were similar to the proportions reported in sub-Saharan Africa (hypertension: 27.9%; aware: 42.7%; controlled: 49.3%), except for the proportion of South Africans receiving treatment for their hypertension which was greater than in sub-Saharan Africa (34.5%) [11]. Although the proportion with hypertension in an opportunistic sample of adults in South Africa was lower than in Europe (43.6%), the mean age in Europe was at least a decade older (50.5 years). Indeed, when age- and sex-standardised proportions were compared, the proportion with hypertension in sub-Saharan Africa did not differ significantly from that in Europe (31.2% and 36.2%, respectively) [11]. However, the proportions of participants with hypertension and receiving treatment for their hypertension and aware of their condition in South Africa were substantially lower than in Europe (64.4 and 71.5%, respectively). The proportion of individuals with hypertension receiving treatment for hypertension is reported to be influenced by country income, with 62% of individuals with hypertension receiving antihypertensive medication in high-income countries compared to only 51.5% in low to middle-income countries [11]. Nevertheless, the proportion of South Africans with hypertension and controlled BP was similar to that in Europe (47.9%) [11].

With regards to age, it is well known that the prevalence of hypertension increases with advancing age. However, it is worth noting that although the current study in an opportunistic sample, shows that 15.6% of young (<40 years of age) adults in South Africa have hypertension, awareness of hypertension among these young participants with hypertension is remarkably low (24.0%). A study of young adults in the United States similarly reported low levels of hypertension awareness in the young (32% in women with hypertension and 25% in men with hypertension men), despite 12% of young women and 27% of young men having hypertension [12]. Although in the National Health and Nutrition Examination Survey (NHANES), the awareness of hypertension was greater than in an opportunistic sample of South Africans, young (<40 years of age) adults tended to have particularly low hypertension awareness (~45%); whereas older (>40 years of age) participants tended to be more aware of their hypertensive status (>70%) [13]. These age discrepancies in awareness have been attributed to younger adults tending to be healthier; being less likely to see doctors on a regular basis; thus, decreasing the likelihood that they will have accurate and up-to-date knowledge of their BP status. In the current study, treatment of hypertension was also low in the young adults (18.6%). These data in an opportunistic sample support similar reports of ~20% of younger adults with hypertension receiving treatment as compared to >55% of older adults with hypertension in a population sample [13]. Therefore, lack of treatment of hypertension and lack of awareness of hypertension seem to be particularly problematic amongst young adults. In order to prevent the long-term sequelae of hypertension it would be pertinent to encourage BP screening and improve awareness of hypertension particularly in young adults.

With respect to gender, the greater prevalence of hypertension in male compared to female South Africans is similar to global data, where up until 80 years of age, both systolic and diastolic BP were higher in males when compared to females [11]. The higher prevalence of hypertension and greater BP values in men compared to women especially prior to menopause are well documented [6, 7, 14, 15]. Mechanisms for the sex differences in hypertension have been linked to various hormonal systems. Oestrogen via the activation of nitric oxide is associated with a lower BP in women [16]. In addition, data obtained in animal models of hypertension suggests that the greater anti-inflammatory immune profile reported in hypertensive females, may act as a compensatory mechanism to limit increases in BP [17]. In comparison, males exhibit a more pro-inflammatory immune profile. Although, the mechanisms underlying these changes in immune cells in hypertensive males and females are not well understood; a possible mediator is the angiotensin type 2 receptor, which promotes an anti-inflammatory immune profile and has a greater activity in females [18].

In addition to sex differences in the prevalence of hypertension, the proportions of hypertensives receiving treatment for their hypertension or aware of their hypertensive status also differed according to sex. In this regard, the current study in an opportunistic sample of adults, showed that treatment and awareness were lower in men compared to women. Discrepancies of treatment and awareness of hypertension between genders have previously been reported. Indeed, a study of young adults in the United States similarly reported lower levels of hypertension awareness in men compared to women, especially amongst those younger than 40 years of age (32% in young hypertensive women compared to 25% in young hypertensive men) [12]. Reports on sex differences in hypertension awareness have produced consistent results, with women having higher levels of hypertension awareness than in men [13, 19, 20]. With regards to BP control in an opportunistic sample of adults, we found that a higher proportion of women had controlled BP compared to men. Similarly, in NHANES from 1999–2014, young adult males were found to have poorer control of hypertension compared to all other age cohorts and age-matched females [21]. The incongruity in BP control according to sex in NHANES, was hypothesised to be due to more frequent healthcare visits by young adult women versus age-matched men [21]. Indeed, globally in MMM, the proportion of individuals reporting never having had their BP measured previously, was higher in men (35.9%) than in women (28.9%) [11]. These data suggest that particular attention should be made to increase screening, awareness and treatment of hypertension in men.

The lack of impact of ethnicity on the prevalence of hypertension in the current study (an opportunistic sample of adults), differs from data in the SANHANES 2011–2012, where hypertension was more prevalent in Indian/Asian (44.9%), Coloured (40.5%), and White (40.4%) participants than in African participants (32.9%), despite similar overall prevalence of hypertension (35.3%) [6]. However, Reddy et al. [6] reported that amongst African participants there was a lower prevalence of hypertension in rural informal compared to urban formal settings. Our data of similar prevalence of hypertension amongst African participants compared to other ethnic groups may be a consequence of screening individuals primarily from urban formal settings. In the current study, the highest prevalence of hypertension was amongst those of mixed ancestry, which agrees with data from the 2016 South African DHS where hypertension was reported to be higher in those of mixed ancestry [7].

In the current study (an opportunistic sample of adults), the highest prevalence of hypertension was observed in the Western Cape, whereas the lowest prevalence was in the North West Province. In the 2016 South African DHS, the highest prevalence was found in KwaZulu Natal with the lowest prevalence in Limpopo [7]. In comparison, in 2012, the SANHANES found that the prevalence of hypertension was the highest in the North West Province and the lowest in Limpopo [6]. The current study also showed provincial differences in the prevalence of hypertension awareness, treatment and BP control. However, neither SANHANES [6] nor the South African DHS [7] reported on hypertension awareness, treatment and BP control.

In line with the United Nations Declaration on the Prevention and Control of Non-Communicable Diseases, in 2022 South Africa developed a National Strategic Plan (NSP) in which they proposed a 90-60-50 cascade for hypertension as the first step to improving early detection and treatment of non-communicable disease [22]. The plan is that by 2030, 90% of all people over 18 years of age will know whether or not they have raised BP, 60% of people with raised BP will receive intervention, and 50% of people receiving interventions will have controlled BP [22]. The data from the current study shows that we are far from realising the South African NSP goal with regard to awareness of hypertension. However, we are closer to the goals of proportions being treated and at least 50% of those treated do have controlled BP. Hence, South Africa needs to increase screening considerably to improve awareness of hypertension and once diagnosed management needs to be instituted.

## Limitations

Although the data in the present study were collected by means of random opportunistic screening and hence not designed to be representative of the whole of South Africa, each year the proportions reported are consistent. Furthermore, our data collected in the MMM campaign in South Africa are in accordance with previous studies (not MMM studies) demonstrating that 30–35% of adults have hypertension, with a high proportion being unaware and a small percentage being treated [23–25]. The diagnosis of hypertension was based upon BP measurements made on only one occasion. However, it has been suggested that if clinic readings are used, the mean of the 2nd and 3rd readings provide conservative estimates of hypertension [11]. Although the decrease in BP values with repeated measurements was reduced by eliminating the 1st reading from the calculation of the mean values, it is still possible that the prevalence of hypertension is an overestimate. Nevertheless, as discussed above, our data are in accordance with previous studies demonstrating that 30–35% of adults have hypertension [23–25]. Measures of the benefits of MMM campaign are difficult to ascertain as only ~7% of participants had previously been involved in an MMM campaign. Furthermore, MMM screening is anonymous and hence follow-up is not possible. However, all of those with untreated hypertension were given dietary and lifestyle advice to lower their BP and referred for further management. In addition, there was coverage in traditional and social media outlets suggestive of improved education and awareness. Nevertheless, MMM remains an inexpensive (investigators are volunteers) but effective means of detecting individuals who require further management of their BP. In addition, MMM serves as a means of raising awareness of hypertension.

## Conclusions

A high prevalence of hypertension and yet low levels of awareness, treatment and BP control are an issue in South Africa, similar to in other countries in sub-Saharan Africa. The low levels of awareness and treatment amongst the young is particularly concerning as are the lower proportions of males with hypertension who are aware of their hypertension and receiving treatment for their hypertension. As the proportions aware of their hypertension status are well below the South African National Strategic Goal of 90%, these data highlight the need for systematic BP screening programmes in South Africa and improvements in education and management of hypertension.

## Summary

### What is known about this topic

- Hypertension is the principal modifiable risk factor for cardiovascular diseases.
- The prevalence of hypertension remains high globally.
- Awareness, treatment and control of blood pressure remain low globally.

### What this study adds

- In South Africa, the prevalence of hypertension is high, but awareness, treatment and blood pressure control are poor.
- The prevalence of hypertension is higher in men than in women, particularly amongst young adults.
- The inadequate awareness, treatment and blood pressure are worse in men than in women and in young adults compared to older individuals.

## Data Availability

Data are not publicly available but are available with permission from the MMM Management Board on request through the MMM website: maymeasure.org.
